# *In Vitro* Characterization of the Pharmacological Properties of the Anti-Cancer Chelator, Bp4eT, and Its Phase I Metabolites

**DOI:** 10.1371/journal.pone.0139929

**Published:** 2015-10-13

**Authors:** Eliška Potůčková, Jaroslav Roh, Miloslav Macháček, Sumit Sahni, Ján Stariat, Vít Šesták, Hana Jansová, Pavlína Hašková, Anna Jirkovská, Kateřina Vávrová, Petra Kovaříková, Danuta S. Kalinowski, Des R. Richardson, Tomáš Šimůnek

**Affiliations:** 1 Department of Biochemical Sciences, Charles University in Prague, Faculty of Pharmacy, Hradec Králové, Czech Republic; 2 Department of Inorganic and Organic Chemistry, Charles University in Prague, Faculty of Pharmacy, Hradec Králové, Czech Republic; 3 Molecular Pharmacology and Pathology Program, Bosch Institute and Department of Pathology, University of Sydney, Sydney, Australia; 4 Department of Pharmaceutical Chemistry and Drug Analysis, Charles University in Prague, Faculty of Pharmacy, Hradec Králové, Czech Republic; University of Melbourne, AUSTRALIA

## Abstract

Cancer cells have a high iron requirement and many experimental studies, as well as clinical trials, have demonstrated that iron chelators are potential anti-cancer agents. The ligand, 2-benzoylpyridine 4-ethyl-3-thiosemicarbazone (Bp4eT), demonstrates both potent anti-neoplastic and anti-retroviral properties. In this study, Bp4eT and its recently identified amidrazone and semicarbazone metabolites were examined and compared with respect to their anti-proliferative activity towards cancer cells (HL-60 human promyelocytic leukemia, MCF-7 human breast adenocarcinoma, HCT116 human colon carcinoma and A549 human lung adenocarcinoma), non-cancerous cells (H9c2 neonatal rat-derived cardiomyoblasts and 3T3 mouse embryo fibroblasts) and their interaction with intracellular iron pools. Bp4eT was demonstrated to be a highly potent and selective anti-neoplastic agent that induces S phase cell cycle arrest, mitochondrial depolarization and apoptosis in MCF-7 cells. Both semicarbazone and amidrazone metabolites showed at least a 300-fold decrease in cytotoxic activity than Bp4eT towards both cancer and normal cell lines. The metabolites also lost the ability to: ***(1)*** promote the redox cycling of iron; ***(2)*** bind and mobilize iron from labile intracellular pools; and ***(3)*** prevent ^59^Fe uptake from ^59^Fe-labeled transferrin by MCF-7 cells. Hence, this study demonstrates that the highly active ligand, Bp4eT, is metabolized to non-toxic and pharmacologically inactive analogs, which most likely contribute to its favorable pharmacological profile. These findings are important for the further development of this drug candidate and contribute to the understanding of the structure-activity relationships of these agents.

## Introduction

Iron is an essential cofactor for the activity of many enzymes crucial for cellular proliferation, including ribonucleotide reductase, which catalyzes the rate-limiting step in DNA synthesis [1]. As cancer cells are generally more metabolically active than their normal counterparts, they require larger amounts of iron [2]. Hence, targeting iron in cancer cells using specific chelators is a promising strategy for the development of novel anti-cancer agents [3]. The thiosemicarbazone class of iron chelators have shown high anti-neoplastic efficiency in both *in vitro* and *in vivo* studies and some agents are also in phase I and II clinical trials [4,5,6,7].

**Fig 1:**
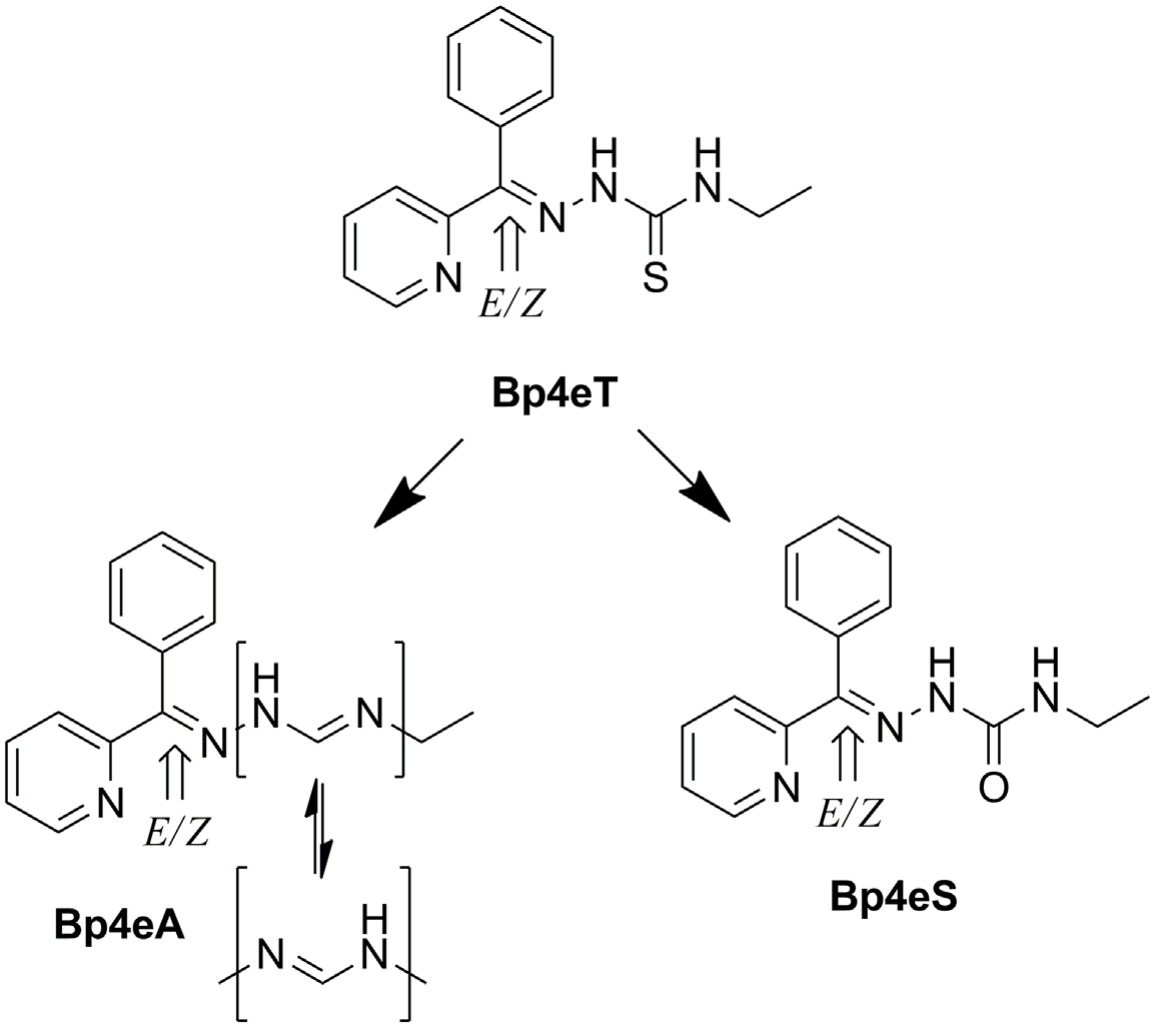
Line drawings of the structures of Bp4eT and its metabolites and indication of *E/Z* isomerism. Bp4eT, 2-benzoylpyridine 4-ethyl-3-thiosemicarbazone; Bp4eA; *N* ^*3*^-ethyl-*N* ^*1*^-[phenyl(pyridine-2yl)methylene]formamidrazone; Bp4eS, 2-benzoylpyridine 4-ethylsemicarbazone.

The ligand, 2-benzoylpyridine 4-ethyl-3-thiosemicarbazone (Bp4eT, Fig 1), was initially synthesized and characterized by West *et al*. [8]. It was later demonstrated to be an iron chelator that possessed a low, positive Fe^3+/2+^ redox potential [9], which resulted in the formation of toxic reactive oxygen species (ROS) both in solution [9] and in cancer cells [10]. In fact, Bp4eT showed high anti-proliferative activity against human SK-N-MC neuroepithelioma cells with low toxicity to normal human MRC-5 fibroblasts [10]. Apart from its anti-cancer activity, Bp4eT showed potent inhibition of HIV-1 transcription with efficacy comparable to that of a clinically used anti-retroviral agent, roscovitin, and exhibited low cytotoxicity in the human T cell lymphoblast-like cell line, CCRF-CEM [11].

In terms of its pharmacokinetics, Bp4eT was shown to easily permeate confluent monolayers of Caco-2 cells, with permeability characteristics similar to common orally administered drugs, indicating bioavailability through this therapeutic route [12,13]. Merlot *et al*. revealed that cellular uptake of ^14^C-Bp4eT in SK-N-MC neuroepithelioma cells was mediated by passive diffusion and that the Fe-Bp4eT complex was sequestered within cells to a greater extent than that of the free Bp4eT ligand [14,15]. Further studies revealed that ^14^C-labeled Bp4eT was excreted quickly from mice *via* the urine and was excreted more slowly *via* the feces, with the main sites of ^14^C-Bp4eT deposition being the organs associated with excretion *e*.*g*., gallbladder, small intestine and large intestine [15].

The metabolism and pharmacokinetics of Bp4eT was further studied in rats using a sensitive LC-MS method [16,17,18]. First, it was demonstrated that Bp4eT existed as a mixture of two interconvertible *E* and *Z* isomers in both aqueous media and plasma, while the *Z* form was predominant in the solid state [16,17,18]. Second, Bp4eT was shown to undergo metabolism *via* oxidation of its thiocarbonyl moiety both *in vitro* and *in vivo*, resulting in the generation of the semicarbazone analog (2-benzoylpyridine 4-ethylsemicarbazone; Bp4eS, Fig 1) and the amidrazone derivative (*N* ^*3*^-ethyl-*N* ^*1*^-[phenyl(pyridin-2-yl)methylene]formamidrazone; Bp4eA, Fig 1) [17]. The amidrazone metabolite was further hydroxylated *in vivo*, but the specific localization of the hydroxyl group on the phenyl ring could not be identified [17].

The Bp4eS metabolite was detected as two *E*/*Z* isomers that were, in contrast to the parent compound, non-interconvertible [18]. Pharmacokinetic investigations revealed that after intravenous administration of Bp4eT, the exposure of rats to the metabolite, Bp4eS, was only minor relative to Bp4eT [18]. On the contrary, the metabolic conversion of administered Bp4eT to the Bp4eA metabolite appeared to be an important biotransformation, as its exposure was 20% of that of the parent compound [18].

Examining the biological properties of drug metabolites is an important step in pharmaceutical development, as the metabolites can significantly contribute to the pharmacological properties of the parent drug [19,20] and may also be of interest for further drug discovery. Hence, to better characterize Bp4eT as a promising drug candidate, we assessed the *in vitro* cytotoxic activities of Bp4eT itself and its two major metabolites, Bp4eA and Bp4eS, on four human cancer cell lines and two non-cancerous cell lines. As iron chelation is a key feature in the mechanism of action of Bp4eT, we examined the ability of Bp4eT and its metabolites to: ***(i)*** bind iron from the labile iron pool (LIP) of cancer cells; ***(ii)*** to mobilize cellular ^59^Fe; and ***(iii)*** prevent the cellular uptake of ^59^Fe from ^59^Fe_2_-transferrin. The ability of the iron complexes of Bp4eT and its metabolites to promote ROS formation was also investigated using the ascorbate oxidation assay. Furthermore, cell cycle progression and the mode of cell death after their exposure to Bp4eT and its metabolites were also determined.

## Materials and Methods

### Chemicals

Bp4eT was synthesized according to Kalinowski *et al*. [9] and its metabolites were synthesized as described by Stariat *et al*. [17,18]. Constituents for various buffers and other chemicals (*e*.*g*., various iron salts) were purchased from Sigma-Aldrich (St. Louis, MO, USA) or Penta (Prague, Czech Republic) and were of the highest pharmaceutical or analytical grade available.

### Cell culture

The human MCF-7 breast adenocarcinoma cell line was purchased from the European Collection of Cell Cultures (ECACC; Salisbury, UK). Human HL-60 promyelocytic leukemia cells, human HCT116 colorectal carcinoma cells, human A549 lung adenocarcinoma cells, the H9c2 cell line, derived from embryonic rat heart tissue, and 3T3 mouse embryo fibroblasts were obtained from the American Type Culture Collection (ATCC; Manassas, VA, USA). The MCF-7, HCT116, A549, 3T3 and H9c2 cell-types were cultured in Dulbecco’s modified Eagle’s medium (DMEM; Lonza, Basel, Switzerland). In the case of MCF-7 cells, DMEM was used without phenol red. DMEM was supplemented with 10% (v/v) heat-inactivated fetal bovine serum (FBS; Lonza), 1% penicillin/streptomycin solution (Lonza) and 10 mM HEPES buffer (pH 7.0–7.6; Sigma-Aldrich). The HL-60 cell line was maintained in RPMI medium (Sigma-Aldrich) supplemented with 10% heat-inactivated FBS and 1% penicillin/streptomycin solution. All cell lines were cultured in 75 cm^2^ tissue culture flasks (TPP, Trasadingen, Switzerland) at 37°C in a humidified atmosphere of 5% CO_2_. Sub-confluent adherent cells, or the suspension of HL-60 cells, were sub-cultured every 3–4 days.

### Cytotoxicity studies

For cytotoxicity experiments, cancer cells were seeded at a density of 5,000 (MCF-7), 10,000 (HL-60) or 2,000 cells/well (HCT116 and A549) in 96-well plates (TPP) for 24 h/37°C prior to the addition of examined agents. The non-cancerous cells, 3T3 and H9c2 cells, were cultured for 24 h/37°C in 96-well plates at a density of 10,000 cells/well, the medium was then changed to serum- and pyruvate-free DMEM (Sigma-Aldrich) and incubated with the cells for another 24 h/37°C. The cytotoxic effects of Bp4eT and its metabolites were studied at different concentrations after a 72 h/37°C incubation. In order to aid the dissolution of the lipophilic ligands, 0.1% dimethyl sulfoxide (v/v) (DMSO; Sigma-Aldrich) was present in the culture medium of all groups. At this concentration, DMSO had no effect on cellular proliferation or viability.

The viability of cells were determined using an MTT assay (Sigma-Aldrich) according to previously established methods [21,22]. The optical density of soluble MTT was measured at λ = 570 nm, subtracting the λ = 690 nm background using a Tecan Infinite 200M plate reader (Tecan Group, Männedorf, Switzerland). The viability or proliferation of experimental groups was expressed as a percentage of the untreated controls (100%).

### Calcein-AM assay for assessment of rate of cell membrane permeation and access to the labile iron pool

These experiments were performed according to Glickstein *et al*. [23] with slight modifications. The MCF-7 cells were seeded in 96-well plates (10,000 cells/well) and allowed to adhere for 24 h/37°C. Cells were loaded with iron using the cellular iron donor, ferric ammonium citrate (530 μg/mL) [24], 24 h prior to the experiment, and then washed. To prevent potential interference, especially with regard to various trace elements, the medium was replaced with ADS buffer (prepared using Millipore water supplemented with 116 mM NaCl, 5.3 mM KCl, 1 mM CaCl_2_, 1.2 mM MgSO_4_, 1.13 mM NaH_2_PO_4_, 5 mM D-glucose, and 20 mM HEPES, pH 7.4). Cells were then loaded with 2 μM of the cell-permeable calcein green acetoxymethyl ester (calcein-AM; Molecular Probes, Oregon, USA) for 30 min/37°C and washed. Cellular esterases cleave the acetoxymethyl groups to form the cell membrane-impermeable compound, calcein green [23]. The fluorescence of calcein green is quenched upon binding iron [23]. The intracellular fluorescence (λ_ex_ = 488 nm; λ_em_ = 530 nm) of calcein green was then followed as a function of time (10 min after the addition of 10 μM Bp4eT or its metabolites) at 37°C using the Tecan Infinite 200M plate reader. The iron chelation efficacy of the metabolites in cells was expressed as a percentage of the efficacy of the parent chelator, Bp4eT (100%).

### Preparation of ^59^Fe_2_-transferrin

Human transferrin (Sigma) was labeled with ^56^Fe or ^59^Fe (PerkinElmer, Massachusetts, USA) to produce ^56^Fe_2_-transferrin or ^59^Fe_2_-transferrin (^59^Fe_2_-Tf), respectively, with a final specific activity of 500 pCi/pmol Fe, as previously described [24,25]. Unbound ^59^Fe was removed by exhaustive vacuum dialysis against a large excess of 0.15 M NaCl buffered to pH 7.4 with 1.4% NaHCO_3_ by standard methods [24,25].

#### The effect of Bp4eT and its metabolites on mobilizing cellular ^59^Fe

To examine the ability of studied compounds to mobilize cellular ^59^Fe from MCF-7 cells, iron efflux experiments were performed using established techniques [21,22]. In brief, after pre-labeling confluent MCF-7 cells on 6-well plates with 0.75 μM ^59^Fe_2_-Tf for 3 h/37°C, the cells were washed four times with ice-cold PBS and then subsequently incubated with 25 μM of Bp4eT or its metabolites for 3 h/37°C. The overlying medium containing released ^59^Fe was then decanted from the cells. Radioactivity was measured in both the cells and the supernatant using a γ-scintillation counter (Wallac Wizard 3, Turku, Finland).

#### Effect of the studied agents on preventing cellular ^59^Fe uptake from ^59^Fe_2_-transferrin

The ability of the chelators to prevent cellular ^59^Fe uptake from ^59^Fe_2_-transferrin was examined using standard techniques [26,27]. In brief, confluent MCF-7 cells in 6-well plates were incubated with 0.75 μM ^59^Fe_2_-Tf for 3 h/37°C in the presence of Bp4eT or its metabolites (25 μM). The cells were then washed four times with ice-cold PBS and the level of internalized ^59^Fe was determined by incubating the cell monolayer for 30 min/4°C with the general protease, Pronase (1 mg/mL; Sigma-Aldrich). The cells were then removed from the monolayer with a plastic spatula on ice and centrifuged for 1 min/12,000 x *g*/4°C. The supernatant represents membrane-bound, Pronase-sensitive ^59^Fe that was released by the protease, while the Pronase-insensitive fraction represents internalized ^59^Fe [21,26,27]. The amount of internalized ^59^Fe was expressed as a percentage of ^59^Fe internalized by control (untreated) cells.

### Ascorbate oxidation assay

The ascorbate oxidation assay was used to assess the redox activity of the iron complexes of the chelators using an established protocol [26,28]. In brief, 100 μM ascorbic acid was prepared immediately prior to the experiment and incubated either alone or in the presence of 10 μM FeCl_3_ in a 50-fold molar excess (500 μM) of citrate and the chelators. Chelators were assayed at iron-binding equivalents (IBE) of 0.1 (excess of iron), 1 (fully coordinated iron—chelator complexes) and 3 (excess of free chelator). The decrease in absorbance at λ = 265 nm was measured after a 10 and 40 min incubation at room temperature using the Tecan Infinite 200M plate reader. The decrease of absorbance between the two time points was calculated and expressed as a percentage of control without the chelator.

### Cell cycle analysis

To examine the effect of the agents on the cell cycle, MCF-7 cells were seeded in 60 mm Petri dishes at a density of 240,000 cells/dish and incubated with Bp4eT or its metabolites for 72 h/37°C. The cells were then harvested, fixed by ethanol and stained by propidium iodide (Molecular Probes, Eugene, OR, USA) for 30 min/37°C, as described previously [29]. Cells were analyzed using Accuri C6 flow cytometer (Becton Dickinson and Company, San Jose, CA USA). Propidium iodide was excited at λ_ex_ = 488 nm and fluorescence analyzed at λ_em_ = 585 nm (FL-2) with a total of 10,000 events collected per analysis.

### Fluorescence microscopy assessments

Markers used to assess autophagy/apoptosis/necrosis in MCF-7 cells and changes of lysosomal and mitochondrial morphology were observed using an Eclipse Ti inverted epifluorescence microscope (Nikon, Tokyo, Japan), that was equipped with a cooled digital camera Zyla 5.5 sCMOS (Andor Technology, Belfast, UK), and NIS-Elements C 4.1 software (Laboratory Imaging, Prague, Czech Republic). The MCF-7 cells were seeded in 6-well plates with cover slips on the bottom at a density of 150,000 cells/well and incubated as described above in the presence or absence of 10 or 100 nM Bp4eT.

To assess the mechanism of cellular death after incubation with Bp4eT, triple staining with monodansyl cadaverine (MDC; 50 μM; λ_ex_ = 390 nm; λ_em_ = 455 nm; Sigma-Aldrich), annexin V-FITC (5 μL/mL; λ_ex_ = 495 nm; λ_em_ = 519 nm; Invitrogen, Carlsbad, CA, USA), and propidium iodide (5 μg/mL; λ_ex_ = 560 nm; λ_em_ = 630 nm) was used. MDC is a marker of autophagosomes and lysosomes and results in blue fluorescence [30,31]. As a positive control for autophagy, MCF-7 cells were incubated with 1 nM rapamycin (Sigma-Aldrich) for 30 min/37°C, which is an established inducer of autophagy [30]. Annexin V has high affinity to phosphatidylserine, which is translocated to the surface of both early- and late-stage apoptotic cells [32,33]. Thus, annexin V-FITC served as a marker of apoptosis when the apoptotic cells had green fluorescent cytoplasmic membranes. Propidium iodide is a necrotic marker, or a marker of late stage apoptosis, as it does not permeate into cells with intact cytoplasmic membranes [34]. The cells were incubated with these probes for 10 min/37°C, washed with fresh cultivation medium and the images captured using the microscope outlined above.

To determine the effect of Bp4eT on mitochondrial morphology, the cells were incubated with MitoTracker^®^ Green FM (0.25 μM; λ_ex_ = 490 nm; λ_em_ = 516 nm; Molecular Probes) for 10 min/37°C. The cells were then washed with fresh medium and the images captured using the microscope described above.

### Western blot analysis

Established protocols were used to prepare cell lysates and perform immunoblot analysis [35]. Primary antibodies used include: rabbit LC3 (Cat. #: MBPM036; 1:2,000) from Abacus (Brisbane, Australia) and mouse β-actin (Cat. #: A1978, 1:10,000) from Sigma-Aldrich. The following secondary antibodies were utilized: horseradish peroxidase (HRP)-conjugated anti-rabbit (Cat. #: A6154, 1:1,0000) and anti-mouse (Cat. #: A4416, 1:10,000) antibodies from Sigma-Aldrich. To ensure equal loading of proteins, membranes were probed for β-actin.

### Caspase activity assessments

To assess the effect of the compounds on caspase activity, MCF-7 cells were incubated with 100 nM Bp4eT or its metabolites for 3, 24 or 72 h/37°C in 96-well plates, as described above. The cells were then lysed by adding 100 μL of cold lysis buffer (100 mM HEPES, 10 mM CHAPS, 10 mM D-L-dithiothreitol, pH 7.4) to 100 μL of medium in each well. Lysates were immediately frozen at -80°C. Thawed lysates were used to assess caspase activity using luminescent kits for caspases 3/7, 8 and 9 (Promega, Madison, WI, USA). The luminescence was measured using the Tecan Infinite 200M plate reader. Caspase activity in the experimental groups was corrected according to the cellular viability of each group and expressed as a percentage of activity of the untreated control (100%).

### Data analysis and statistics

SigmaStat for Windows 3.5 (Systat Software, San Jose, CA, USA) statistical software package was utilized to analyze results. The data are expressed as the mean ± SD of a given number of experiments. Statistical significance was determined using a one-way ANOVA with a Bonferroni *post-hoc* test or Student’s *t*-test. The results were considered to be statistically significant when *p* < 0.05. The IC_50_ values were calculated using CalcuSyn 2.0 software (Biosoft, Cambridge, UK). Cell cycle analysis was evaluated using MultiCycle AV Software (Phoenix Flow Systems, San Diego, CA, USA).

## Results and Discussion

### Bp4eT is metabolized into compounds with at least a 300-fold decrease in cytotoxicity against both cancer and non-cancerous cells

The cytotoxic activity of Bp4eT was compared to Bp4eA (used as a mixture of *E* and *Z* isomers) and Bp4eS (in two isomeric forms: *E-*Bp4eS and *Z-*Bp4eS). The *E* and *Z* isomers of Bp4eS were examined separately, as they were both detected *in vivo* in previous studies [18], and thus, are biologically significant. However, these two isomers are not interconvertible and are separate compounds that can be isolated and analyzed [18]. In contrast, Bp4eA readily interconverts between the *E* and *Z* isomeric states [18], and due to this inherent physical property, only the mixture of these isomers can be assessed. In these studies, the effects of the agents on cancer cells were studied using human HL-60 promyelocytic leukemia, human MCF-7 breast adenocarcinoma, human HCT116 colorectal carcinoma and human A549 lung adenocarcinoma cell lines, as well as two non-cancerous cell-types, namely rat H9c2 cardiomyoblasts, and mouse 3T3 fibroblasts.

**Fig 2:**
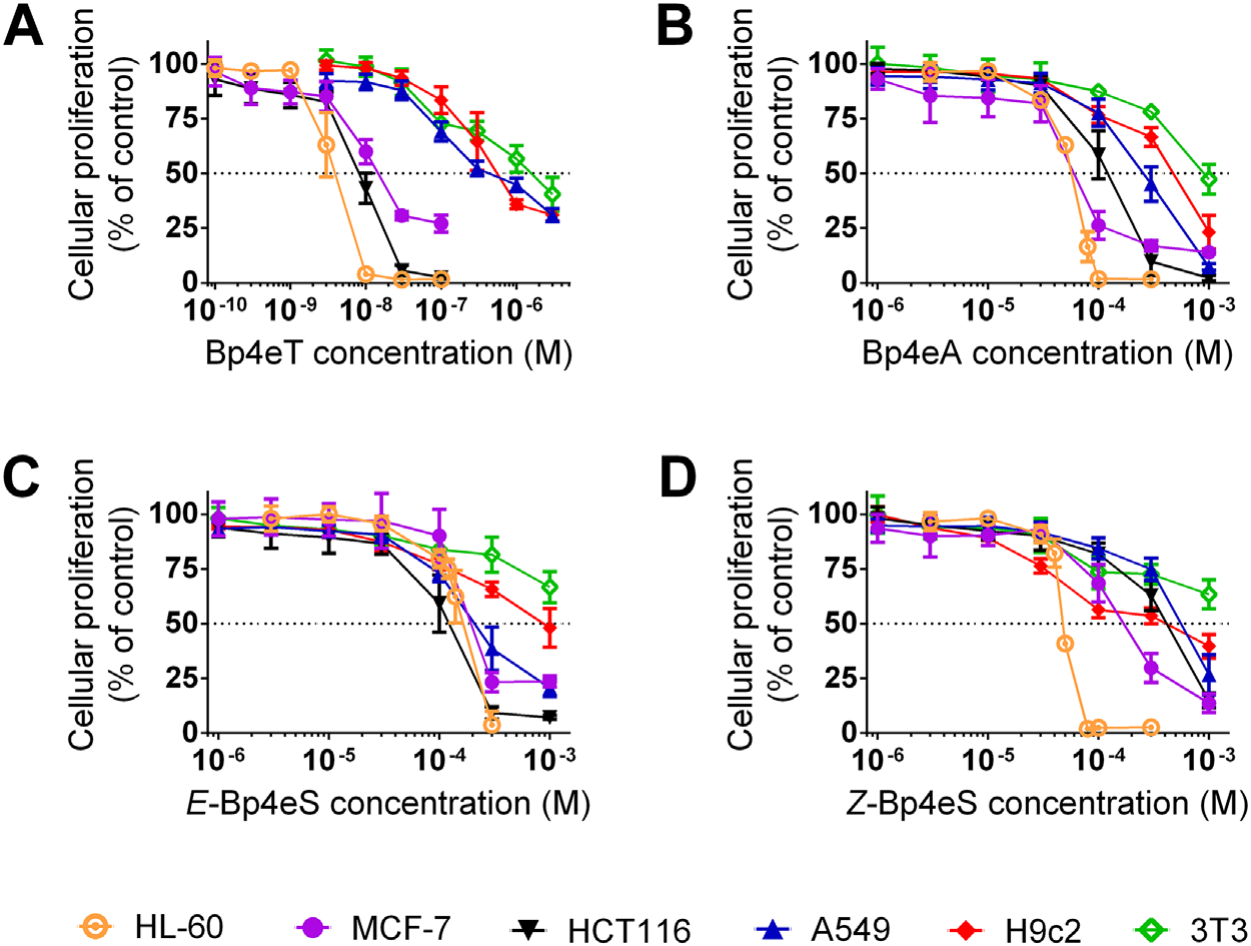
Anti-proliferative activity of Bp4eT (A) and its metabolites, Bp4eA (B), and both *E* (C) and *Z* (D) isomers of Bp4eS. For determination of anti-proliferative activity, the cancer cell lines (*i*.*e*., HL-60, MCF-7, HCT116 and A549) and non-cancer cell lines (*i*.*e*., H9c2 and 3T3) were incubated with the agents for 72 h/37°C and proliferation then assessed using the MTT assay. The results are mean ± SD (*n* ≥ 4 experiments).

Following a 72 h incubation, the parent compound, Bp4eT, showed very potent cytotoxic effects against HL-60, MCF-7 and HCT116 cells, where the IC_50_ values ranged from 3 to 15 nM (Table 1 and Fig 2A). The anti-cancer activity of Bp4eT towards these cell lines was markedly greater than that of the clinically used chelators, deferoxamine or deferasirox, which have IC_50_ values in the μM range against cancer cells [9,36,37,38]. The IC_50_ value of Bp4eT against A549 cells was moderate (IC_50_ = 0.593 ± 0.148 μM) and was comparable to the cytotoxic effects of Bp4eT against H9c2 cardiomyoblasts (IC_50_ = 0.524 ± 0.157 μM; Table 1). The IC_50_ value of Bp4eT against 3T3 fibroblast cells (IC_50_ = 1.309 ± 0.337 μM; Table 1) was two-fold greater than that observed with H9c2 cells. In fact, 3T3 fibroblasts were the most resistant of all the cell-types to every agent examined. Moreover, the cytotoxicity of Bp4eT against 3T3 fibroblast cells was similar to that observed previously against human MRC-5 fibroblast cells, with IC_50_ values ranging from 0.7 to >6 μM [9,10,39].

**Table 1:** Cytotoxic effects of Bp4eT and its metabolites against both neoplastic and non-cancerous cell lines.

	IC_50_ (μM)					
	HL-60	MCF-7	HCT116	A549	H9c2	3T3
Bp4eT	0.003 ± 0.001	0.015 ± 0.002	0.008 ± 0.001	0.593 ± 0.148	0.524 ± 0.157	1.309 ± 0.337
Bp4eA	52.1 ± 3.3	59.4 ± 8.9	111.5 ± 20.9	206.8 ± 46.1	416.1 ± 122.1	1027.4 ± 203.9
*E*-Bp4eS	150.6 ± 9.1	208.5 ± 43.9	95.4 ± 10.4	247.9 ± 28.4	883.8 ± 278.6	>1000
*Z*-Bp4eS	46.2 ± 1.5	197.6 ± 20.9	337.0 ± 48.5	535.9 ± 147.2	343.2 ± 95.1	>1000

Bp4eT and its metabolites were incubated with HL-60, MCF-7, HCT116 and A549 cancer cells or H9c2 and 3T3 non-cancerous cells at 37°C/72 h. Cellular viability was determined using the MTT assay and the IC_50_ values (half-maximal inhibitory concentrations) were calculated using CalcuSyn 2.0 software. Mean ± SD; *n* ≥ 4 experiments.

The therapeutic index was calculated by dividing the IC_50_ in normal cells by the IC_50_ obtained in neoplastic cells and this index acted as a measure of the selectivity of the agent towards cancer cells (Table 2). Importantly, the therapeutic indices of Bp4eT towards HL-60, MCF-7 and HCT116 cancer cells were high (34.9–436.3; Table 2), indicating the selectivity of Bp4eT against these cancer-types. This is in agreement with previous studies demonstrating the potent and selective anti-neoplastic activity of Bp4eT [9,40]. As described above, the selectivity of Bp4eT against A549 cells was low, resulting in therapeutic indices of 0.9–2.2 (Table 2).

**Table 2:** Therapeutic indices of Bp4eT and its metabolites against neoplastic cells.

	IC_50_ non-cancerous cells / IC_50_ neoplastic cells							
	H9c2/HL-60	3T3/HL-60	H9c2/MCF-7	3T3/MCF-7	H9c2/HCT116	3T3/HCT116	H9c2/A549	3T3/A549
Bp4eT	174.7	436.3	34.9	87.3	65.5	163.6	0.9	2.2
Bp4eA	8.0	19.7	7.0	17.3	3.7	9.2	2.0	5.0
*E*-Bp4eS	5.9	>6.6	4.2	>4.8	9.3	>10.5	3.6	>4.0
*Z*-Bp4eS	7.4	>21.6	1.7	>5.1	1.0	>3.0	0.6	>1.9

The therapeutic indices were calculated using the following ratio, IC_50_ non-cancerous cells / IC_50_ neoplastic cells. Results are means of *n* ≥ 4 experiments.

The Bp4eT metabolites demonstrated a marked decrease in cytotoxicity against both cancer and non-cancerous cell lines (Table 1 and Fig 2) and their IC_50_ values were >300-fold higher relative to the parent agent. The amidrazone, Bp4eA, which was identified as the major metabolite of Bp4eT [18], was generally more cytotoxic against cancer cells (with the exception of HCT116 cells) than the semicarbazone metabolite, Bp4eS. The IC_50_ values of Bp4eA against cancer cells ranged from 52 to 207 μM (Table 1). Additionally, the cytotoxicity of Bp4eA against non-cancerous cells was lower in comparison to the cancer cells examined, with IC_50_ values of 416.1 ± 122.1 μM and 1027.4 ± 203.9 μM for H9c2 and 3T3 cells, respectively. This was also reflected in the therapeutic indices of Bp4eA, which ranged from 2.0 to 19.7 (Table 2). Importantly, the toxic concentrations of Bp4eA against normal cells were not reached in plasma during our previous pharmacokinetic study, where the highest concentration of Bp4eA reached was < 1 μM after 300 min post *i*.*v*. administration of Bp4eT [18]. This clearly suggests that Bp4eA levels in plasma were at non-toxic concentrations.

Both the non-interconvertible *E* and *Z* isomers of the Bp4eS metabolite were previously identified at low concentrations (< 0.02 μM) in plasma [18]. Our results demonstrated that Bp4eS generally had poorer anti-proliferative activity than Bp4eA (Table 1 and Fig 2), with IC_50_ values ranging between 46 to 536 μM in cancer cells and 343 μM and >1 mM in non-cancerous H9c2 and 3T3 cells, respectively. Surprisingly, each cell line showed differential sensitivity to the *E* and *Z* isomers of Bp4eS. Although HL-60 and H9c2 cells were significantly (*p*<0.001) more sensitive to the *Z* isomer, HCT116 and A549 cells were significantly (*p*<0.01) more sensitive to *E* isomer of Bp4eS (Table 1). In contrast, MCF-7 cells were approximately equally sensitive to both the *E* and *Z* isomers of Bp4eS (Table 1). Relative to Bp4eT, the therapeutic indices of the *E* and *Z* isomers of Bp4eS were generally low, especially against H9c2 cells and ranged from 0.6 to >21.6 (Table 2). As the *E* and *Z* isomers of Bp4eS were only detected at very low concentrations in plasma [18], and since their cytotoxic effects occur only at high concentrations, it can be suggested that Bp4eS would show low anti-proliferative activity against cancer cells and toxicity to normal cells *in vivo*.

### The ability of Bp4eT metabolites to chelate iron from the labile iron pool, mobilize cellular ^59^Fe and prevent cellular ^59^Fe uptake from ^59^Fe_2_-transferrin is negligible compared to Bp4eT

**Fig 3:**
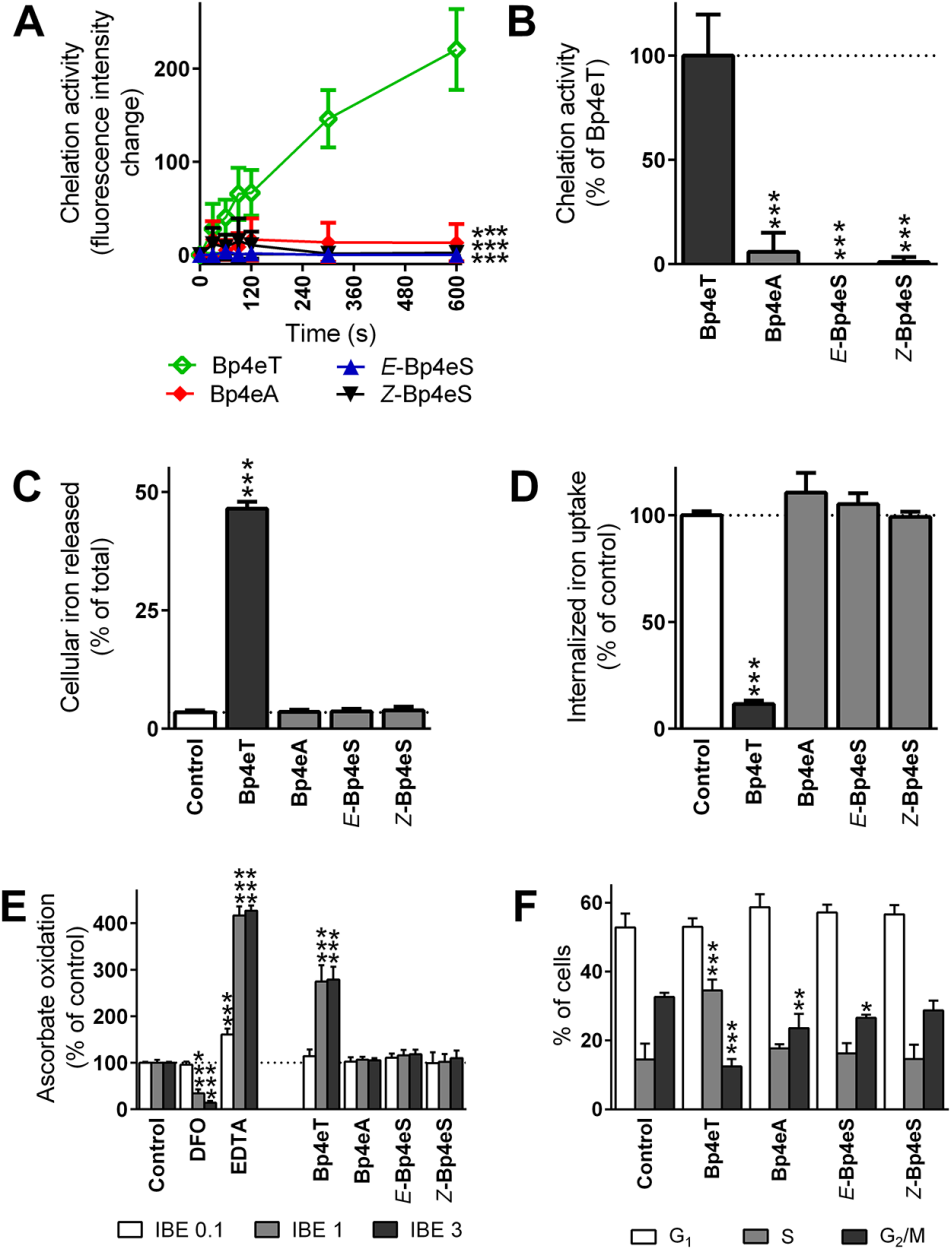
The ability of Bp4eA and Bp4eS to bind iron, to form redox active iron complexes and to cause an increase in the S phase and a decrease of G_2_/M phase of cell cycle was negligible compared to the parent chelator, Bp4eT. The efficacy of Bp4eT or its metabolites to chelate iron from the LIP of MCF-7 cells was measured using the calcein-AM assay. (A) Fluorescence of free calcein after the addition of 10 μM Bp4eT or its metabolites for 10 min/37°C. (B) Intensity of fluorescence of free calcein in the presence of the metabolites at *t* = 600 s was expressed as a percentage of the fluorescence of free calcein in the presence of Bp4eT. The results of (A) and (B) are mean ± SD (*n* = 6 experiments). Statistical significance (ANOVA): *** *p* < 0.001 as compared to Bp4eT. (C) Efflux of ^59^Fe mediated by control medium or medium containing the agents (25 μM) after a 3 h/37°C incubation of MCF-7 cells prelabeled with ^59^Fe-transferrin. (D) Uptake of ^59^Fe from ^59^Fe_2_-transferrin by MCF-7 cells in the presence of control medium or medium containing the agents (25 μM) were determined after a 3 h/37°C incubation. The results of (C) and (D) are mean ± SD (*n* ≥ 3 experiments). Statistical significance (ANOVA): *** *p* < 0.001 as compared to the control (untreated) group. (E) The ascorbate oxidation assay was used to examine the formation of redox active complexes. Bp4eT and its metabolites were assayed at iron binding equivalents (IBE) of 0.1 (excess of iron to chelator); 1 (fully complete coordination shell); and 3 (excess of chelator to iron). The chelators, DFO and EDTA, were used as anti-oxidative or pro-oxidative controls, respectively. Data are expressed as a percentage of the control group without chelator at the same IBE (100%). The results of (E) are mean ± SD (*n* ≥ 3) experiments. Statistical significance (ANOVA): *** *p* < 0.001 as compared to the control group (iron with ascorbate) in the same IBE. (F) MCF-7 cells were incubated for 72 h/37°C with 100 nM of Bp4eT or its metabolites, Bp4eA and Bp4eS. Cell cycle analysis was processed by flow cytometry using propidium iodide. Phase quantification was evaluated using MultiCycle AV Software. The results of (F) are mean ± SD (*n* ≥ 3 experiments). Statistical significance (ANOVA): * *p* < 0.05, ** *p* < 0.01, *** *p* < 0.001 as compared to the control group.

The ability of Bp4eT and its metabolites to chelate iron from the LIP in MCF-7 cells was investigated in this study using the calcein-AM assay, as iron chelation and depletion are believed to play a role in the anti-cancer activity of the thiosemicarbazones [3,9]. The parent compound, Bp4eT (10 μM), showed a time-dependent increase in fluorescence, due to the ability of Bp4eT to chelate iron from calcein-AM in MCF-7 cells (Fig 3A). In contrast, the addition of the Bp4eT metabolites had almost no effect on calcein-AM fluorescence (Fig 3A). When expressed as a percentage of Bp4eT fluorescence at *t* = 600 s, the metabolite, Bp4eA, showed only 5.9% of the chelation efficacy of Bp4eT, while both isomers of Bp4eS demonstrated ≤1.0% of the fluorescence of Bp4eT (Fig 3B).

Furthermore, Bp4eT demonstrated high ^59^Fe mobilization efficacy and was able to mediate the release of 46.4% of total cellular ^59^Fe (Fig 3C). Neither of the Bp4eT metabolites resulted in a significant release of cellular ^59^Fe and were comparable to the untreated control (3.5% of total ^59^Fe; Fig 3C).

The ability of Bp4eT and its metabolites to prevent the cellular uptake of ^59^Fe from ^59^Fe_2_-transferrin after a 3 h/37°C incubation was also examined in MCF-7 cells. Importantly, the parent chelator, Bp4eT, demonstrated high ^59^Fe chelation efficacy and inhibited internalized ^59^Fe uptake to 11.5% of the control (Fig 3D). As observed in the ^59^Fe efflux assay, both Bp4eA and Bp4eS showed poor ^59^Fe chelation efficacy and their ability to inhibit ^59^Fe uptake was comparable to the untreated control (Fig 3D).

Collectively, the results of these experiments suggested that the metabolites of Bp4eT demonstrated poor iron chelation efficacy. Only the parent, Bp4eT, contains the thioamide group that readily tautomerizes to an imidothiol moiety, allowing the sulfur atom to coordinate with iron (S1 Fig). Iron is additionally coordinated through the pyridine nitrogen and aldimine nitrogen atom in Bp4eT resulting in tridentate ligation, with two Bp4eT chelator being required to complete the coordination shell of the iron atom.

The reason for the low chelation efficacy of the semicarbazone, Bp4eS, could be because the amide moiety highly prevails over the imidol tautomer [41]. Thus, the sulfur atom in the parent thiosemicarbazone, Bp4eT, acts as a better donor atom than the carbonyl oxygen of Bp4eS (S1 Fig).

The metabolite, Bp4eA, contains the formamidrazone moiety and does not possess the sulfur atom of the original thiosemicarbazone, which appears to be crucial in terms of the iron chelation efficacy of Bp4eT (S1 Fig). In addition, the formation of the amidrazone analog results in electron delocalization along the backbone, as the imine double bond is in conjugation with the aldimine double bond. As a result, this delocalization of electrons hinders the ability of the amidrazone metabolite, Bp4eA, to coordinate iron. Additionally, when considering the second possible tautomer of the formamidrazone moiety, this would result in the formation of three weak coordination bonds upon the chelation of iron (S1 Fig).

### Bp4eT metabolites do not form redox-active iron complexes

It has been demonstrated that redox activity of the thiosemicarbazone iron complexes plays a role in the anti-cancer activity of these compounds [9,10,26,42]. Hence, the redox activity of the iron complexes of Bp4eT and its metabolites was examined using the ascorbate oxidation assay (Fig 3E). The effect of Bp4eT and its metabolites on the oxidation of ascorbate in the presence of iron was assayed at three IBEs (0.1; 1 and 3), as per our standard protocol [26,28]. An IBE of 0.1 represents an excess of iron relative to the chelator. An IBE of 1 results in the formation of a fully coordinated iron complex, representing 1 molecule of a hexadentate chelator (*e*.*g*., DFO or EDTA) for 1 atom of iron, or two molecules of a tridentate chelator (*e*.*g*., Bp4eT) for 1 atom of iron. Additionally, an IBE of 3 represents an excess of the chelator relative to iron. The resulting change in the absorbance of ascorbate was expressed as percentage of the control (ascorbate with “free” iron).

Two well-known chelators, DFO and EDTA, were also assessed in this study as negative and positive controls, respectively [26,43]. As previously observed [26,43], DFO demonstrated an anti-oxidant profile, resulting in a significant (*p* < 0.001) decrease in the oxidation of ascorbate at an IBE of 1 and 3 (Fig 3E). In contrast, the positive control, EDTA, significantly (*p* < 0.001) increased ascorbate oxidation to 161, 417 and 427% of the control at IBEs of 0.1, 1 and 3, respectively (Fig 3E). The parent compound, Bp4eT, mediated a significant (*p* < 0.001) increase in the oxidation of ascorbate at IBEs of 1 and 3 (Fig 3E), as previously observed [9]. In contrast, the metabolites of Bp4eT did not mediate the oxidation of ascorbate at all IBEs and were comparable to the control. Hence, these results are in agreement with our iron chelation efficacy studies above, suggesting that unlike Bp4eT which binds iron to form a redox active iron complex [9], the Bp4eT metabolites have limited ability to bind iron, and thus, do not lead to ascorbate oxidation.

### Bp4eT results in cell cycle arrest in the S phase

Iron deprivation is known to cause G_1_/S cell cycle arrest in rapidly proliferating cancer cells [2,44]. Therefore, we analyzed the effect of Bp4eT and its metabolites (0.1 μM) on the cell cycle of MCF-7 cells after a 72 h incubation. This concentration of Bp4eT was utilized as it led to a decrease in MCF-7 proliferation to 27% of the control (Fig 2A).

Interestingly, the G_1_ phase of the cell cycle was not significantly (*p* > 0.05) different from the control after incubation with Bp4eT or its metabolites (Fig 3F). However, after incubation with Bp4eT, the percentage of cells in the S phase was significantly (*p* < 0.001) increased to 35% relative to the control (15%; Fig 3F). Additionally, the percentage of cells in the G_2_/M phase of the cell cycle were significantly (*p* < 0.001) decreased to 12% upon incubation with Bp4eT relative to the control (33%; Fig 3F). This observation suggests that MCF-7 cells were arrested in the S phase of the cell cycle upon incubation with Bp4eT, which is consistent with our previous studies with other iron chelators in MCF-7 cells [45]. Interestingly, the Bp4eT metabolites did not alter the ratio of cells in each phase, except for a slight, but significant (*p* < 0.01–0.05) decrease in cells in the G_2_/M phase upon incubation with Bp4eA and *E*-Bp4eS (Fig 3F).

### Prolonged incubation of MCF-7 cells with Bp4eT suppresses autophagy

Fluorescence microscopy was used to assess the predominant mechanism(s) (*e*.*g*., autophagy, apoptosis and necrosis) involved in the death of MCF-7 cells after incubation with Bp4eT. The Bp4eT metabolites were not investigated in these studies due to their limited cytotoxicity (Table 1). Triple staining utilized: **(1)** the probe, monodansylcadaverine (MDC; blue fluorescence), to examine lysosomes/autophagic vacuoles; **(2)** annexin V-FITC conjugate (green fluorescence) to assess apoptosis [32,33]; and **(3)** the chromatin dye, propidium iodide (red fluorescence), to determine the presence of necrosis [34].

**Fig 4:**
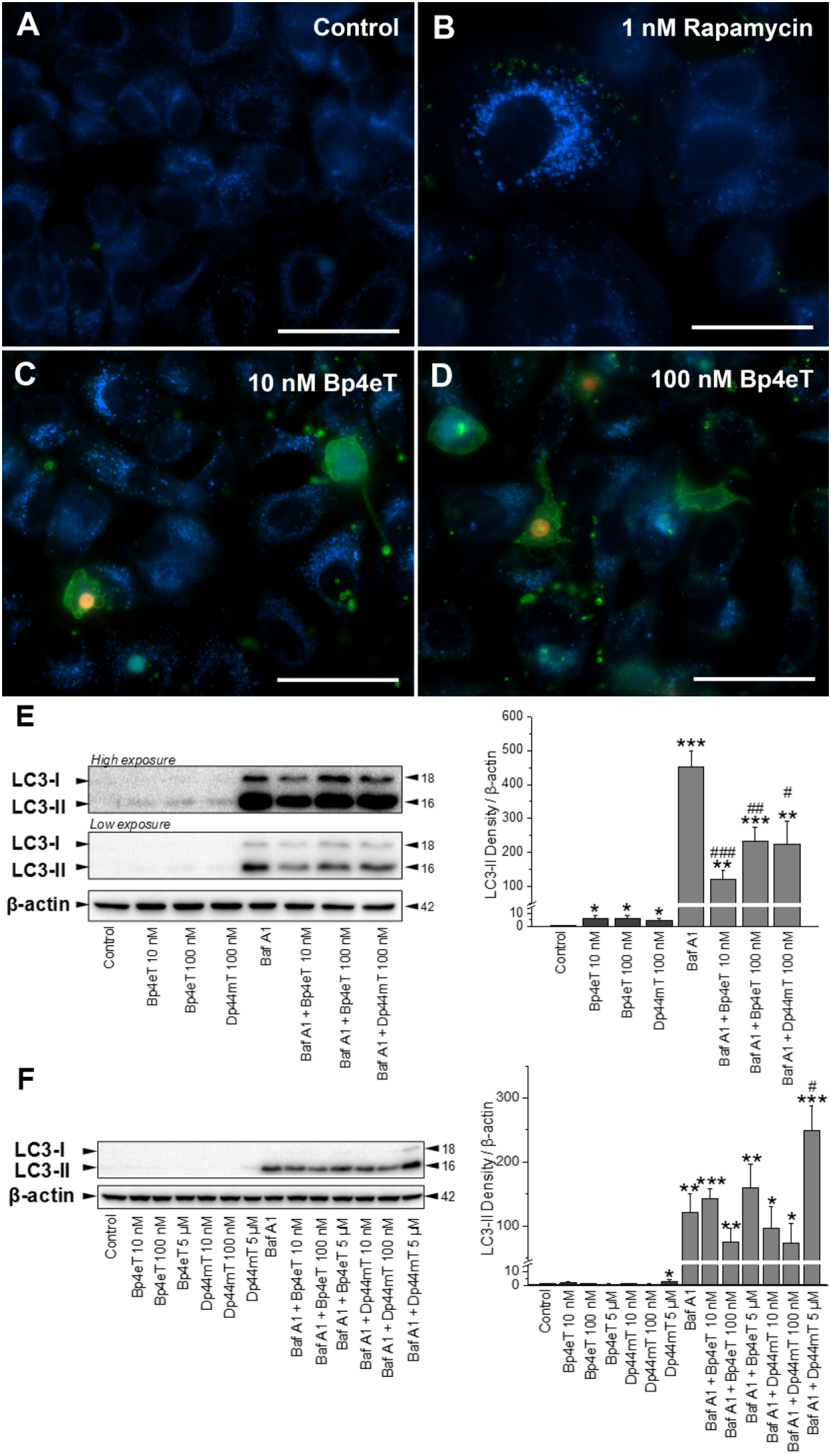
Epifluorescence microscopy and immunoblot estimation of autophagy, apoptosis, or necrosis after incubation with Bp4eT. MCF-7 cells were incubated at 37°C with either: (A) Control medium for 72 h; (B) Rapamycin (1 nM) for 30 min to serve as a positive control for autophagy; or (C, D) 10 or 100 nM Bp4eT for 72 h. Scale bars represent 50 μm. (E) MCF-7 cells were incubated for 72 h/37°C with either: control medium, or medium containing 10 or 100 nM Bp4eT, 100 nM Dp44mT, or 100 nM Bafilomycin A1 alone, or the combination of chelators and Bafilomycin A1. Western blotting and subsequently densitometry was then performed to assess LC3-I/II expression. As there was a marked difference in ability of Bafilomycin A1 and the chelators to up-regulate LC3-II levels, the blots are shown at both low and high exposures. (F) MCF-7 cells were incubated for 24 h/37°C with either: control medium or this medium containing either: Bp4eT (10 nM– 5 μM), Dp44mT (10 nM– 5 μM), or Bafilomycin A1 (100 nM) alone, or the combination of the chelators and Bafilomycin A1. Western blotting and densitometry were then performed. The western analysis in (E) and (F) are typical from 3 experiments, while the densitometric analysis is mean ± SD (3 experiments) normalized to β-actin. *p < 0.05, **p < 0.01, ***p < 0.001 *versus* control. ^#^ *p* < 0.05, ^##^ *p* < 0.01, ^###^ *p* < 0.001 *versus* Bafilomycin A1 alone.

Epifluorescence microscopy of MCF7 cells incubated with control medium for 72 h revealed perinuclear blue punctate staining with MDC, which is known to accumulate in autophagic and lysosomal vacuoles (Fig 4A). Incubation of cells for 30 min with 1 nM rapamycin, a positive control known to induce autophagy [30], led to formation of enlarged cells with ample granular, cytoplasmic MDC staining suggestive of autophagosome formation [46,47] (Fig 4B). A 72 h incubation with 10 nM Bp4eT (Fig 4C) or 100 nM Bp4eT (Fig 4D), resulted in a dose-dependent increase in the number of green annexin V-stained cell membranes or bodies, which is an indication of apoptosis [32,33]. The pale red-fluorescent nuclei stained with propidium iodide (Fig 4D) or intense yellow nuclei when co-localized with green fluorescence of cell membranes (Fig 4C and 4D) are nuclei of necrotic or late-stage apoptotic cells with altered cell membrane integrity [34]. Notably, an increase in blue punctate fluorescence was also observed relative to the control, but it was difficult to determine whether this represented lysosomes, autophagic vacuoles, or a mixture of both (Fig 4C and 4D). Hence, further studies were performed to assess the induction of autophagy by Bp4eT, particularly as recent investigations using the same cell-type and a related thiosemicarbazone, namely Dp44mT, demonstrated induction of this process [48].

In order to additionally examine the effect of Bp4eT on autophagy, immunoblot analysis of the well characterized autophagy marker, LC3-II, was performed [49,50]. The levels of cellular LC3-II corresponds to the number of autophagosomes, and thus, it is a suitable and well characterized marker to assess autophagy [49,50]. Moreover, as autophagy is a dynamic process, the levels of LC3-II observed can be due to either increased autophagic initiation (autophagosome formation), or to decreased autophagic degradation (lysosome-mediated breakdown of autophagosomes) [49,50]. To ascertain which of these mechanisms was involved in the Bp4eT-mediated effects on autophagy, we further incubated cells with the late-stage autophagic inhibitor, Bafilomycin A1 (Baf A1), in the presence or absence of the thiosemicarbazones [48],[50]. Baf A1 is known to inhibit autophagic degradation *via* two pathways: **(1)** inhibition of lysosome-autophagosome fusion; and **(2)** prevention of lysosomal acidification [50].

MCF-7 cells were incubated with Bp4eT (10 and 100 nM) in the presence or absence of Baf A1 (100 nM) for 72 h/37°C (Fig 4E). In these studies, Dp44mT (100 nM) was employed as a relevant positive control as it has been previously shown to induce autophagy at 5 μM after a 24 h incubation [48,51]. Immunoblot analysis was then performed of proteins extracted from cells incubated under different conditions (Fig 4E). As there was a marked difference in ability of Baf A1 and the thiosemicarbazones to up-regulate LC3-II levels, the blots are shown at both low and high exposures in order to demonstrate their effect on LC3-II levels (Fig 4E). When cells were incubated with Bp4eT or Dp44mT alone, there was a slight, but significant (*p* < 0.05) increase in LC3-II levels compared to the control (see High exposure LC3-I/II blot; Fig 4E). Incubation with Baf A1 alone under control conditions led to a marked and significant (*p* < 0.001) increase in LC3-II levels relative to the control without Baf A1 (Fig 4E; low exposure LC3-I/II blot). The level of LC3-II after incubation with Baf A1 alone represents the basal autophagic flux in the cell [50]. Furthermore, upon co-incubation of cells with Baf A1 and either Bp4eT or Dp44mT, a significant (*p* < 0.001–0.01) increase in LC3-II levels was observed compared to the control without Baf A1 (low exposure LC3-I/II blot; Fig 4E). However, there was significant (*p* < 0.001–0.05) suppression in LC3-II levels upon co-incubation of Baf A1 and thiosemicarbazones compared to Baf A1 alone (low exposure LC3-I/II blot; Fig 4E). This observation indicates that both Bp4eT and Dp44mT suppress autophagic initiation under these conditions. These results are different to the previously reported induction of the autophagic initiation pathway by Dp44mT [48,51]. However, in those previous studies, Dp44mT was employed at much a higher concentration (5 μM) for a shorter period of time (24 h). These conditions were in contrast to the present investigation where a lower dose (100 nM) was utilized over a prolonged incubation (72 h).

In order to directly compare our results to these earlier reports [48,51], MCF-7 cells were then incubated for 24 h/37°C with Bp4eT (10 nM– 5 μM) or Dp44mT (10 nM– 5 μM) in the presence or absence of Baf A1 (100 nM; Fig 4F). In agreement with previous studies, we observed that Dp44mT and Baf A1 (5 μM) led to a significant (*p* < 0.05) increase in LC3-II after a 24 h incubation relative to Baf A1 and control (Fig 4F). This observation in the presence of Baf A1 indicates an increase in the autophagic flux. However, Baf A1 and Dp44mT at lower concentrations (10 and 100 nM), as well as Baf A1 and Bp4eT at all concentrations (10 nM– 5 μM), did not lead to a significant (*p* > 0.05) increase in LC3II expression relative to Baf A1 and control (Fig 4F). The observed increase in autophagic initiation by Dp44mT (5 μM/24 h/37°C) may be explained by an initial response of the cell to the stress induced by this agent.

With respect to these studies, Sahni *et al*. have previously shown that the metastasis suppressor, NDRG1, which is molecular target of Bp4eT and Dp44mT, can suppress the autophagic initiation pathway [51]. Hence, it can be speculated that the observed suppression in LC3II levels, and thus, autophagic initiation after a prolonged incubation (72 h) with Bp4eT and Dp44mT in the presence of Baf A1 (Fig 4A), is due to the up-regulation of NDRG1 *via* these agents that then suppresses autophagy [51]. Collectively, it can be concluded that prolonged incubations of Bp4eT resulted in suppression of the autophagic pathway. As autophagy is known to play a survival role in the cellular stress response [52], suppression of this pathway by Bp4eT may make cells more susceptible to death induced by apoptosis and/or necrosis.

### Bp4eT alters mitochondrial morphology and induces apoptosis

**Fig 5:**
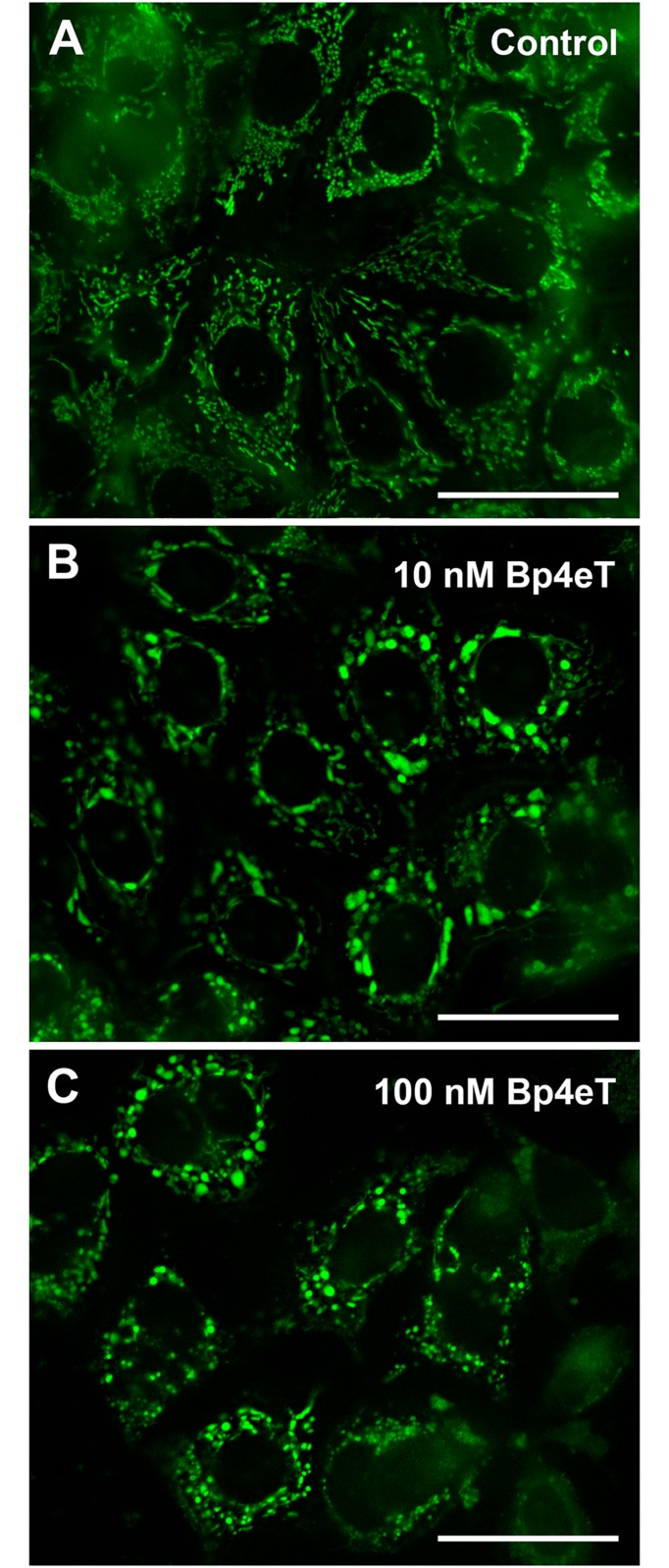
Bp4eT treatment causes mitochondrial swelling. MCF-7 cells were incubated for 72 h/37°C with control medium or 10 or 100 nM Bp4eT, followed by staining for 10 min/37°C with the mitochondrial probe, MitoTracker^®^ Green FM. Scale bars represent 50 μm. Results are typical of 3 experiments.

To additionally examine the mode of cell death induced by Bp4eT (10 or 100 nM) after a 72 h incubation, staining of mitochondria with MitoTracker^®^ Green FM was implemented (Fig 5). Mitochondria are predominantly depolarized and disrupted following the activation of the intrinsic apoptotic pathway [53]. As seen in Fig 5A, intracellular structures consistent with the mitochondria of control cells were detected as green rod-shaped or filamentous particles. In contrast, upon incubation with 10 or 100 nM Bp4eT (Fig 5B and 5C) there was a marked alteration in mitochondrial morphology, where these organelles appeared more swollen and enlarged. Hence, these results demonstrate alterations in mitochondrial morphology after incubation with Bp4eT suggesting the possible role of this organelle in the anti-proliferative activity of this agent.

**Fig 6:**
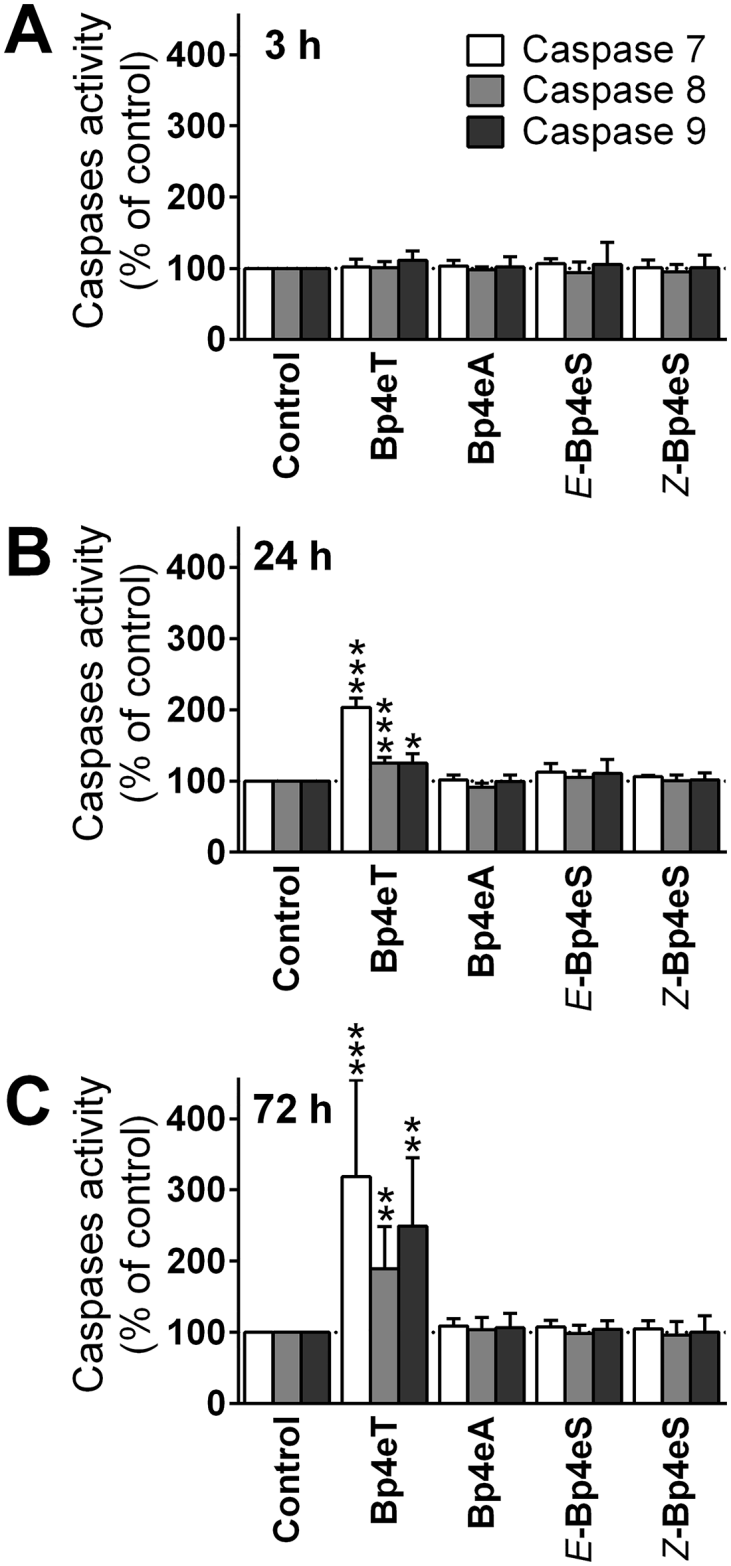
Incubation of Bp4eT with MCF-7 cells caused increased caspase activity, whereas its metabolites did not. MCF-7 cells were incubated for: (A) 3 h; (B) 24 h; or (C) 72 h/37°C with 100 nM Bp4eT or its metabolites, Bp4eA and Bp4eS. The caspase activities were then assayed in cellular lysates. The activities were related to cell viabilities and the results were expressed as a percentage of control. The results are mean ± SD (*n* = 4 experiments). Statistical significance (ANOVA): * *p* < 0.05, ** *p* < 0.01, *** *p* < 0.001 as compared to the control (untreated) group.

To further investigate the effect of Bp4eT and its metabolites with respect to the activation of apoptosis, we examined their effect on the activities of caspases, which are key enzymes in apoptotic signaling [54]. The activity of the effector caspase 7, the extrinsic apoptotic pathway caspase 8, and the intrinsic apoptotic pathway caspase 9, were measured after 3, 24 and 72 h incubations of MCF-7 cells with Bp4eT or its metabolites (100 nM; Fig 6A–6C). Caspase 3 activity was not considered as MCF-7 cells are known to lack caspase 3 expression and it is bypassed in the apoptotic cascade [55].

None of the Bp4eT metabolites were able to activate any of the examined caspases, at all time points, indicating that they were unable to induce apoptosis at the concentration used (Fig 6A–6C). On the other hand, while the parent chelator, Bp4eT, did not activate any of the caspases after a 3 h incubation (Fig 6A), an increase in caspase activity was observed after 24 h and 72 h incubations (Fig 6B and 6C). The activity of both the initiator caspases, namely caspase 8 and 9, were significantly (*p* < 0.001–0.05) increased to 125% of the control after a 24 h incubation, and to 189% (caspase 8) and 249% (caspase 9) of the control after 72 h (Fig 6). These observations suggest the activation of both the intrinsic and extrinsic apoptotic pathways by Bp4eT. The activity of the effector caspase 7 demonstrated the greatest increase of all caspases examined (Fig 6B and 6C). In fact, its activity was significantly (*p* < 0.001) increased to 203% of the control after a 24 h incubation, and to 318% of control after a 72 h incubation with Bp4eT (Fig 6B and 6C).

## Conclusions

The results of this study show that Bp4eT is a highly potent and selective anti-neoplastic agent that causes S phase cell cycle arrest, suppression of autophagy, mitochondrial swelling and apoptotic cell death. The metabolic conversion of the thiosemicarbazone group of Bp4eT to the amidrazone or semicarbazone moiety leads to diminished iron chelation and mobilization activity, loss of redox activity of the iron complexes and a two order of magnitude reduction of anti-proliferative activity and toxicity. Hence, the Bp4eT metabolites do not contribute to its pharmacological activity. The findings of this investigation are of importance for further development of this group of novel anti-cancer thiosemicarbazones.

## Supporting Information

S1 Data Raw Experimental Data 1st Part. (ZIP)

S2 Data Raw Experimental Data 2nd Part. (ZIP)

S1 Fig Expected iron complexes of Bp4eT and its metabolites. (TIF)
