# Supplementary figures and images for: In Vitro Characterization of the Pharmacological Properties of the Anti-Cancer Chelator, Bp4eT, and Its Phase I Metabolites
Source: PLoS One. 2015 Oct 13;10(10):e0139929. doi: 10.1371/journal.pone.0139929 (PMC4604124; doi:10.1371/journal.pone.0139929)

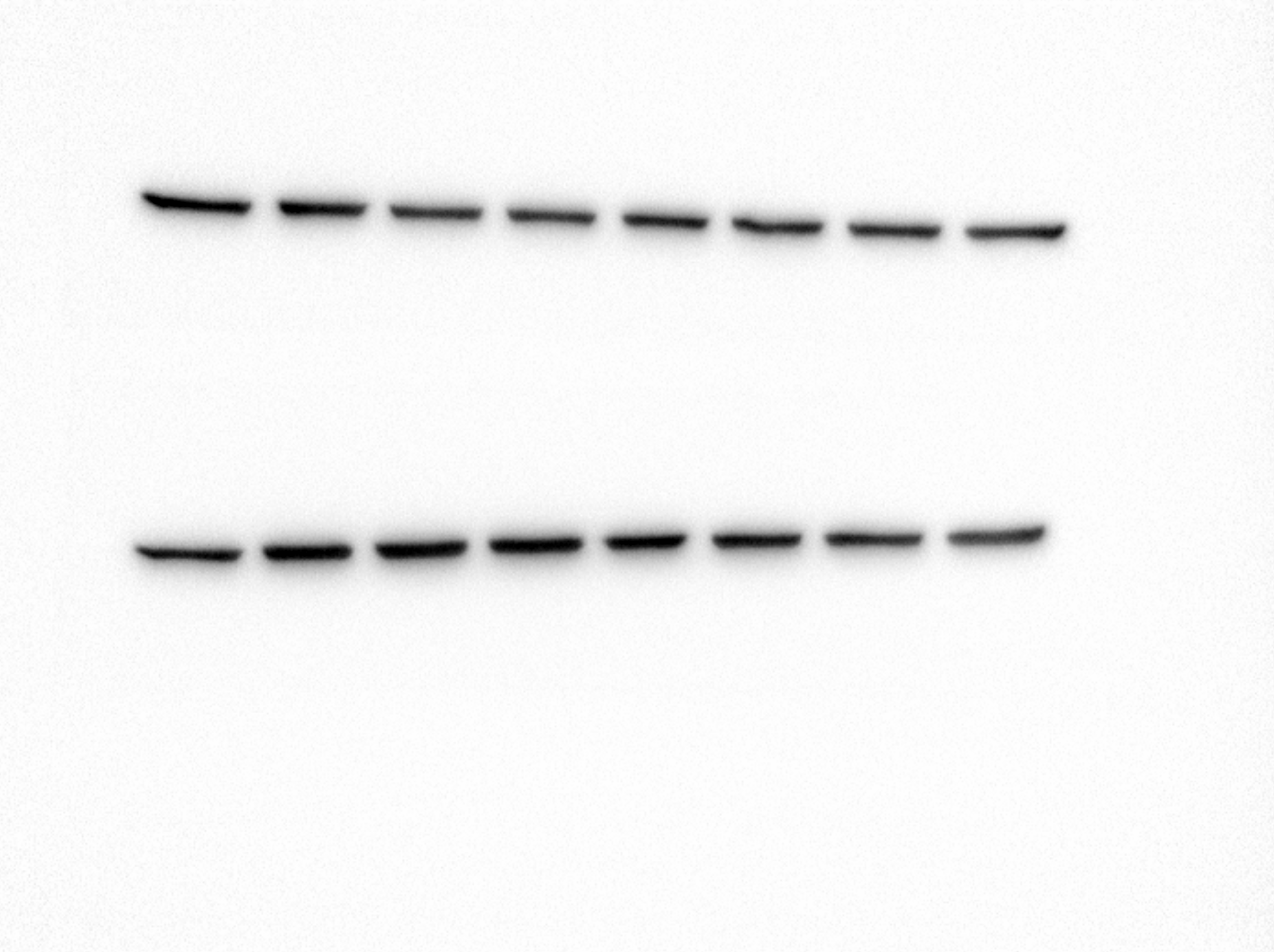

Supplement: S2 Data — (ZIP) [file pone.0139929.s002.zip › B-actin_Bottom Blot.tif]

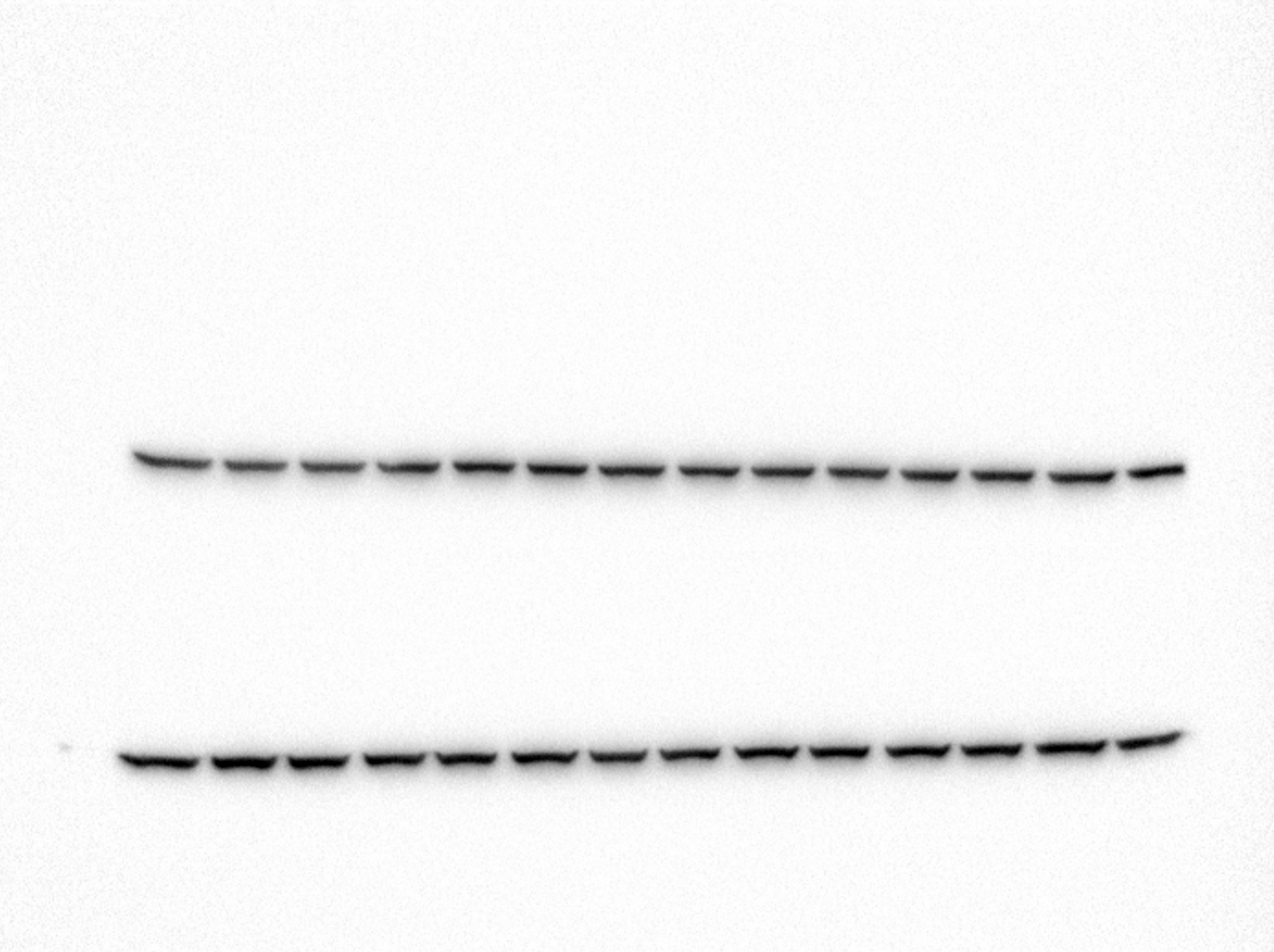

Supplement: S2 Data — (ZIP) [file pone.0139929.s002.zip › B-actin_Bottom Blot2.tif]

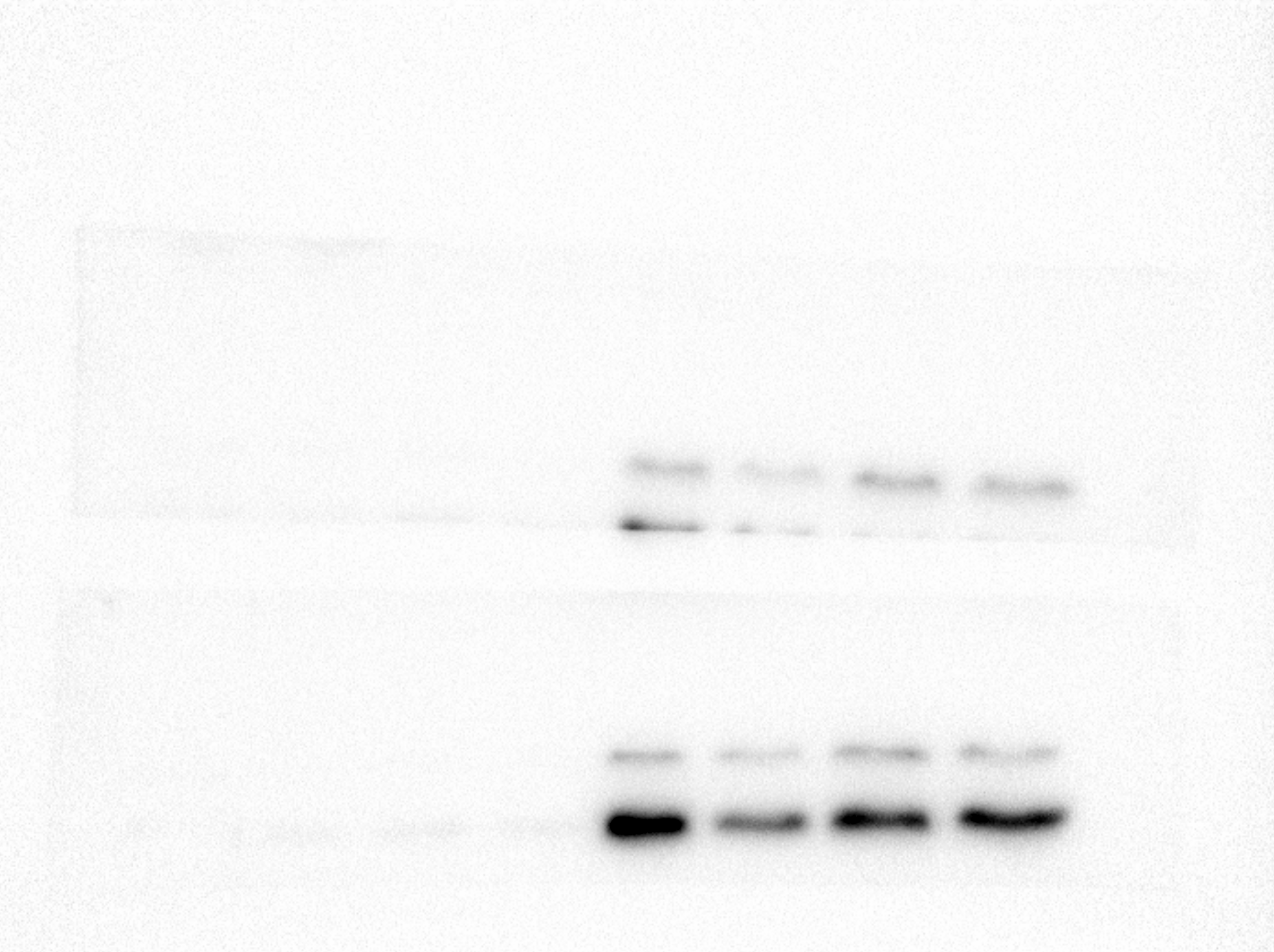

Supplement: S2 Data — (ZIP) [file pone.0139929.s002.zip › LC3_Bottom Blot.tif]

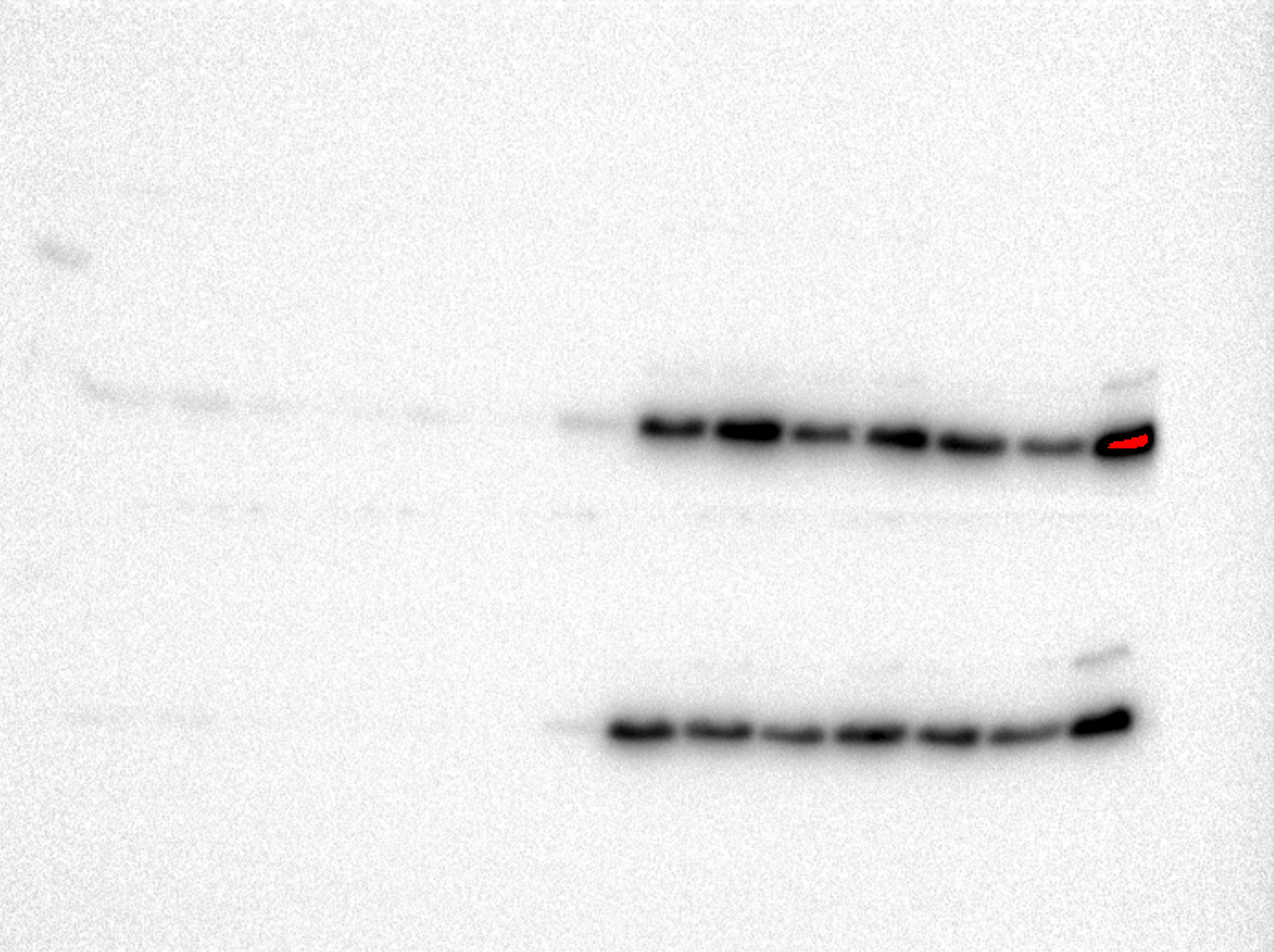

Supplement: S2 Data — (ZIP) [file pone.0139929.s002.zip › LC3_Bottom Blot2.tif]
